# Maternal concern and refusal toward neonatal heel-prick screening: a cross-sectional survey from Türkiye

**DOI:** 10.1007/s00431-026-07025-y

**Published:** 2026-05-07

**Authors:** Hatice Turgut, Gülseda Boz, Tuba Güven, Ramazan Özdemir

**Affiliations:** 1 Department of Pediatrics, Division of Neonatology, Faculty of Medicine, İnönü University, Malatya, Turkey; 2 Department of Public Health, Faculty of Medicine, İnönü University, Malatya, Turkey; 3 Department of Pediatrics, Faculty of Medicine, İnönü University, Malatya, Turkey

**Keywords:** Heel-prick screening, Dried-blood-spot testing, Maternal concern, Neonatal screening

## Abstract

Neonatal heel-prick screening is a key component of preventive healthcare, and its effectiveness is strongly influenced by parents’ knowledge, perceptions, and willingness to engage in screening. The aim of this study was to evaluate the relationships between mothers’ sociodemographic characteristics and their attitudes and behaviors toward the screening process and the level of concern they experience regarding newborn screening. This cross-sectional study included 350 mothers. Data on sociodemographic characteristics and knowledge, attitudes, and behaviors regarding neonatal heel-prick screening were collected using a researcher-developed structured questionnaire. Maternal concern, defined as a self-reported binary outcome (present/absent), was the primary outcome variable. Associations were analyzed using the chi-square test or Fisher’s exact test, as appropriate, and variables with *p* < 0.10 in univariable analyses were entered into a multivariable Firth’s penalized logistic regression model. The results are presented as adjusted odds ratios (aORs) with 95% confidence intervals (CIs), and a two-sided *p* value < 0.05 was considered to indicate statistical significance. Among 350 mothers, 17.7% reported concern related to the heel-prick test, and 9.7% refused the procedure. Concern was more common among those who refused the test (73.5% vs. 11.7%, *p* < 0.001) and those with vaccine concern (42.5% vs. 5.9%, *p* < 0.001). According to the results of the multivariable analysis, vaccine concern was the strongest independent predictor (aOR = 8.98; 95% CI: 4.56–18.69; *p* < 0.001), whereas rural residence (aOR = 3.18; *p* = 0.032) and perceiving the procedure as painful (aOR = 4.70; *p* < 0.001) were also significant risk factors.

*Conclusion*: This study revealed that maternal concern about heel-prick screening was associated with insufficient knowledge, negative perceptions of the procedure, concerns about the vaccine, rural residence, and cultural factors. These findings suggest that clear communication, effective education, and culturally sensitive counselling may help reduce maternal concern and improve participation in newborn screening programs.

**Table:** 

**What is Known:** • *Parental knowledge, perceptions, and communication with healthcare professionals are important determinants of participation in newborn screening programs.* **What is New:** • *Maternal concern toward neonatal heel-prick screening was independently associated with vaccine concern, perceived procedural pain, and rural residence, highlighting the need for targeted and culturally sensitive counselling.*

**Supplementary information:**

The online version contains supplementary material available at 10.1007/s00431-026-07025-y.

## Introduction

Newborn heel-prick screening is a globally implemented program of critical importance to public health. The primary aim of the program is to detect genetic, metabolic, and endocrine disorders at an early stage and ensure that they are treated in a timely manner. Early diagnosis and intervention play key roles in reducing infant mortality, disease-related complications, and long-term healthcare costs, thereby contributing significantly to improved health outcomes and overall quality of life [1–4].

The diseases included in newborn screening programs can vary depending on the epidemiological characteristics of each country or region. Although the scope of screened disorders differs across countries, the fundamental principles and objectives of neonatal screening programs remain consistent worldwide [5, 6].

In Türkiye, newborn heel-prick screening involves taking the first blood sample within the first 48–72 h after birth. A second sample is then taken between 3 and 5 days of life. Within the scope of the screening program, phenylketonuria, congenital hypothyroidism, biotinidase deficiency, cystic fibrosis, congenital adrenal hyperplasia, and spinal muscular atrophy are routinely evaluated. Implementation is carried out in combination with primary healthcare institutions. In the national guidelines, the timing and method of sample collection, laboratory analysis procedures, evaluation of suspected cases, and referral processes are described in detail [7].

The success of newborn screening programs depends not only on the infrastructure of laboratories and the healthcare system but also on the maintenance of strong coordination at every stage of this multi-step process, including the timing of screening, evaluation and reporting of results, and effective communication with families [8]. Moreover, the active participation of parents is a critical factor in ensuring the effectiveness of newborn screening systems. A lack of knowledge, misconceptions, increased parental concern, and negative attitudes toward screening among parents can adversely affect participation rates and, consequently, the overall success of the program. Recent findings suggest that parents generally have insufficient levels of knowledge regarding newborn screening programs [3, 4, 9–11]. On the other hand, it has been reported that parents with greater awareness and knowledge about screening programs demonstrate higher compliance with the implementation of tests [12, 13].

Parents’ beliefs, perceptions, and knowledge about newborn screening are directly associated with the quality and scope of the information provided during the education and counselling process. A comprehensive and effective educational approach improves parents’ understanding of newborn screening programs and contributes to a more positive overall experience [11, 14–16].

Considering the crucial role of parental awareness and participation in the success of newborn screening programs, this study aims to evaluate the relationships among Turkish mothers’ sociodemographic characteristics, their knowledge and perceptions of the screening process, and their levels of concern related to newborn screening. The findings are expected to contribute to the development of strategies aimed at improving informational processes and enhancing parental involvement in newborn screening programs.

## Materials and methods

### Study design and population

This cross-sectional study was conducted between January and June 2025 at İnönü University Turgut Özal Medical Center, a tertiary university hospital located in Malatya, eastern Türkiye. The hospital serves as a regional referral center for both urban and rural populations and provides obstetric, neonatal intensive care, and neonatal outpatient services. Participants were selected using a consecutive sampling method. All postpartum mothers who presented to clinical settings related to newborn care, including the obstetric ward, the neonatal intensive care unit, and the neonatal outpatient clinic, during the study period and met the inclusion criteria were invited to participate. Mothers were included if they were aged 18 years or older, had a newborn eligible for neonatal heel-prick screening, were able to understand and complete the questionnaire, and provided written informed consent. Mothers who declined participation, had severe communication difficulties, or were clinically unable to complete the questionnaire were excluded.

The sample size was calculated using the formula for an unknown population. Assuming a 95% confidence level, a 5% margin of error, and a prevalence of 50%, the minimum required sample size was calculated as 384 participants. During the study period, the study was completed with 350 postpartum mothers who met the inclusion criteria, agreed to participate, and completed the questionnaire. Therefore, the final sample corresponded to 91.1% of the calculated minimum sample size.

### Data collection

Data were collected using a structured questionnaire developed by the researchers on the basis of a comprehensive review of the national and international literature on newborn screening, parental knowledge, attitudes, and procedure-related concerns. Because the questionnaire was specifically designed for the purposes of this study, it was not based on a previously validated psychometric scale, and no formal psychometric validation study was conducted.

The questionnaire consisted of three main sections. The first section included questions assessing the sociodemographic characteristics of the participating mothers, such as age, educational status, occupation, and income level, as well as their obstetric features, including the number of pregnancies, mode of delivery, and antenatal follow-up status. The second section contained questions related to maternal concern regarding neonatal heel-prick screening, including the presence of concern, perceived reasons for concern, and emotional responses associated with the procedure. Maternal concern was assessed as a screening-specific self-reported construct and was operationalized as the mother’s self-reported concern about the heel-prick screening procedure. For the purposes of analysis, this variable was categorized as present or absent. The third section included questions evaluating participants’ knowledge, attitudes, and practices regarding neonatal heel-prick screening. Data were obtained through supervised face-to-face interviews.

### Data analysis

Statistical analyses were performed using IBM SPSS Statistics for Windows, version 25.0 (IBM Corp., Armonk, NY, USA), and R (version 4.5.2; R Foundation for Statistical Computing, Vienna, Austria) was used for multivariable modelling. Descriptive data are presented as frequencies (*n*) and percentages (%). In this study, the dependent variable was maternal concern regarding neonatal heel-prick screening (present/absent), while the independent variables included sociodemographic characteristics and mothers’ knowledge, attitudes, and behaviors toward the screening process. Associations between the dependent variable and categorical independent variables were evaluated using the chi-square test or Fisher’s exact test, as appropriate. Variables found to be statistically significant or approaching significance (*p* < 0.10) in univariable analyses were entered into the multivariable model to identify independent predictors of maternal concern. To account for the relatively low number of events and to minimize small-sample bias, Firth’s penalized logistic regression was used for multivariable analysis. Findings are presented as adjusted odds ratios (aORs) with 95% confidence intervals (CIs). A two-sided *p* value < 0.05 was considered to indicate statistical significance.

### Ethical considerations

Written permission to conduct the study was obtained from the Chief Physician’s Office of İnönü University Turgut Özal Medical Center, and ethical approval was granted by the institutional Scientific Research and Publication Ethics Committee (Approval No: 2025/6841). The study was conducted in accordance with the Declaration of Helsinki. Before the questionnaire was administered, all participants were informed about the purpose of the study, the voluntary nature of participation, the scientific use of the collected data, and the confidentiality of personal information. Written informed consent was obtained from all participants before data collection.

## Results

A total of 350 postpartum mothers were included in the final analysis. Among the participants, 17.7% (*n* = 62) reported concerns regarding heel-prick screening, whereas 82.3% (*n* = 288) expressed no concerns. Additionally, 90.3% (*n* = 316) accepted the heel-prick screening program, whereas 9.7% (*n* = 34) refused the procedure. The median age of the participants was 30 years (range, 19–50). Comparisons of participant characteristics according to maternal concern toward neonatal heel-prick screening are presented in Table 1. No statistically significant differences were found between concern related to heel-prick screening and variables such as age, educational level, or economic status. However, when evaluated by place of residence, a greater percentage of mothers living in rural villages (31.6%) reported concern than those living in other residential areas did (*p* = 0.049). A statistically significant association was found between concern and the decision to allow heel-prick sampling at the current birth (*p* < 0.001). The rate of concern was significantly greater among mothers who declined the heel-prick test (73.5%) than among those who did (11.7%). Mothers who knew someone who had declined heel-prick screening for any reason presented significantly higher levels of concern (43.2%) than those who did not (14.7%) (*p* < 0.001). Similarly, compared with mothers who did not know such individuals (14.7%), mothers who knew someone who had refused heel-prick screening for religious or cultural reasons presented a markedly higher concern rate (50.0%) (*p* < 0.001). The relationship between concern regarding heel-prick screening and hesitancy toward childhood vaccinations was examined. Mothers who reported vaccine concern had a significantly higher rate of concern than those without vaccine concern did (42.5% vs. 5.9%, *p* < 0.001).

**Table 1:** Comparison of participant characteristics by maternal concern toward neonatal heel-prick screening

Variable	Study group (*n* = 350) *n* (%)*	Concern: yes (*n* = 62) *n* (%)**	Concern: no (*n* = 288) *n* (%)**	*p*
Age group				
20–29	161 (46.0)	27 (16.8)	134 (83.2)	
30–39	171 (48.9)	31 (18.1)	140 (81.9)	0.831
≥ 40	18 (5.1)	4 (22.2)	14 (77.8)	
Educational status				
Illiterate	7 (2.0)	0 (0.0)	7 (100.0)	
Primary school	29 (8.3)	5 (17.2)	24 (82.8)	
Secondary school	58 (16.6)	9 (15.5)	49 (84.5)	0.733
High school	107 (30.6)	19 (17.8)	88 (82.2)	
University or higher	149 (42.6)	29 (19.5)	120 (80.5)	
Place of residence				
City center	230 (65.7)	35 (15.2)	195 (84.8)	
District	82 (23.4)	15 (18.3)	67 (81.7)	0.049
Village	38 (10.9)	12 (31.6)	26 (68.4)	
Economic status				
Good	118 (33.7)	18 (15.3)	100 (84.7)	
Moderate	225 (64.3)	43 (19.1)	182 (80.9)	0.655
Poor	7 (2.0)	1 (14.3)	6 (85.7)	
Heel-prick performed in current birth				
Yes	316 (90.3)	37 (11.7)	280 (88.3)	< 0.001
No	34 (9.7)	25 (73.5)	9 (26.5)	
Informed before heel-prick				
Yes	214 (61.1)	33 (15.4)	181 (84.6)	0.159
No	136 (38.9)	29 (21.3)	107 (78.7)	
Knowing someone diagnosed from heel-prick				
Yes	19 (5.4)	5 (26.3)	14 (73.7)	0.351
No	331 (94.6)	57 (17.2)	274 (82.8)	
Knowing someone who refused heel-prick				
Yes	37 (10.6)	16 (43.2)	21 (56.8)	< 0.001
No	313 (89.4)	46 (14.7)	267 (85.3)	
Knowing someone who refused for religious/cultural reasons				
Yes	30 (8.6)	15 (50.0)	15 (50.0)	< 0.001
No	320 (91.4)	47 (14.7)	273 (85.3)	
Vaccine concern				
Yes	113 (32.3)	48 (42.5)	65 (57.5)	< 0.001
No	237 (67.7)	14 (5.9)	223 (94.1)	

*Column percentage, **row percentage

Data are presented as *n* (%). The *χ*^2^ test or Fisher’s exact test was used, as appropriate. *p* < 0.05 was considered to indicate statistical significance

The participants’ perceptions and beliefs regarding the heel-prick blood-sampling procedure were compared according to the presence of concern, as presented in Table 2. The prevalence of concern was 28.8% among mothers who believed the procedure to be painful, whereas it was 7.8% among those who did not share this opinion (*p* < 0.001). Similarly, concern was prevalent among 26.4% of mothers who thought they had not been adequately informed about the heel-prick test, whereas 11.6% of those who believed that sufficient information had been provided (*p* = 0.003) were not. The prevalence of concern was 66.7% among mothers who believed that heel-prick blood sampling was unnecessary, whereas it was 9.6% among those who disagreed with this view (*p* < 0.001). Similarly, 56.6% of mothers who expressed concerns about the reliability of the procedure reported concern, compared with 7.3% of those who considered it safe (*p* < 0.001). Furthermore, concern was present in 66.7% of mothers who believed that heel-prick blood sampling contradicted their religious beliefs, whereas it was present in 15.4% of those who disagreed with this view (*p* < 0.001).

**Table 2:** Evaluation of participants’ perceptions and beliefs about heel-prick screening

Participants’ attitudes and beliefs	Concern: yes (*n* = 62) *n* (%)*	Concern: no (*n* = 288) *n* (%)*	*p*
I think heel-prick sampling is a painful procedure			
Yes^a^ No^b^ Undecided	42 (28.8) 12 (7.8) 8 (16)	104 (71.2) 142 (92.2) 42 (84)	< 0.001
I believe that insufficient information is provided about heel-prick screening			
Yes^a^ No^b^ Undecided	33 (26.4) 22 (11.6) 7 (19.4)	92 (73.6) 167 (88.4) 29 (80.6)	0.003
I think heel-prick sampling is unnecessary			
Yes^a^ No^b^ Undecided	14 (66.7) 28 (9.6) 20 (54.1)	7 (33.3) 264 (90.4) 17 (45.9)	< 0.001
I have concerns about the reliability of heel-prick screening			
Yes^a^ No^b^ Undecided^c^	30 (56.6) 18 (7.3) 14 (28.6)	23 (43.4) 230 (92.7) 35 (71.4)	< 0.001
I am not confident in the effectiveness of heel-prick screening			
Yes^a^ No^b^ Undecided^c^	24 (53.3) 18 (8) 20 (24.7)	21 (46.7) 206 (92) 61 (75.3)	< 0.001
I believe that test results are not communicated to families			
Yes^a^ No^b^ Undecided	25 (36.8) 16 (9.2) 21 (19.3)	43 (63.2) 157 (90.8) 88 (80.7)	< 0.001
I think heel-prick screening is against my religious beliefs			
Yes^a^ No^b^ Undecided^c^	4 (66.7) 49 (15.4) 9 (36)	2 (33.3) 270 (84.6) 16 (64)	< 0.001

^a,b,c^Different from each other, *Row percentage

The participants’ levels of knowledge and awareness of the heel-prick screening test were compared according to their concern status (Table 3). The prevalence of concern was 11.4% among individuals who knew that the heel-prick test was performed to detect genetic and congenital diseases, 21.4% among those who did not know, and 36.8% among those who were uncertain (*p* < 0.001). These findings indicate that mothers who were aware of the purpose of the test experienced significantly less concern.

**Table 3:** Comparison of participants’ knowledge regarding neonatal heel-prick screening by concern status

Variable	Concern: yes (*n* = 62) *n* (%)*	Concern: no (*n* = 288) *n* (%)*	*p*
Knowing which diseases are screened			
Yes No Undecided	28 (18.9) 25 (15) 9 (25.7)	120 (81.1) 142 (85) 26 (74.3)	0.280
Knowing whether screening is legally mandatory			
Yes No Undecided	9 (10.8) 26 (21) 27 (18.9)	74 (89.2) 98 (79) 116 (81.1)	0.156
Thinking screening should be mandatory			
Yes^a^ No^b^ Undecided	9 (6.2) 36 (30.5) 17 (19.5)	136 (93.8) 82 (69.5) 70 (80.5)	< 0.001
Considering refusal as child neglect			
Yes^a^ No^b^ Undecided^c^	12 (7.1) 29 (26.1) 21 (30.4)	158 (92.9) 82 (73.9) 48 (69.6)	< 0.001
Knowing possible risks if screening is not done			
Yes^a^ No^b^ Undecided^c^	22 (17.1) 28 (16.3) 12 (24.5)	107 (82.9) 144 (83.7) 37 (75.5)	0.402
Screening detects genetic/congenital diseases			
Yes^a^ No Undecided^c^	28 (11.4) 6 (21.4) 28 (36.8)	218 (88.6) 22 (78.6) 48 (63.2)	< 0.001
Thinking infant screening only includes heel-prick			
Yes^a^ No^b^ Undecided^c^	8 (12.7) 20 (12.8) 34 (26)	55 (87.3) 136 (87.2) 97 (74)	0.008
Family informed when test is normal			
Yes^a^ No Undecided^c^	31 (14.5) 13 (14.6) 18 (38.3)	183 (85.5) 76 (85.4) 29 (61.7)	< 0.001
Family informed when test is abnormal			
Yes^a^ No Undecided^c^	46 (15.3) 1 (7.7) 15 (40.5)	254 (84.7) 12 (92.3) 22 (59.5)	< 0.001
Results available within 1 week			
Yes^a^ No^b^ Undecided	13 (10.2) 16 (27.1) 33 (20.4)	115 (89.8) 43 (72.9) 129 (79.6)	0.009

^a,b,c^Different from each other, *Row percentage

Awareness of the communication of test results to families was also significantly associated with maternal concern. Among those who believed that families were informed that the test results were normal, the prevalence of concern was 14.5%, whereas it was 38.3% among those who were uncertain (*p* < 0.001). Similarly, concern was present in 15.3% of the participants who agreed that families were informed when the test result was abnormal, but this percentage increased to 40.5% among those who were undecided (*p* < 0.001).

The factors associated with maternal concern toward neonatal heel-prick screening were evaluated through progressive multivariable models. While the sociodemographic distribution of the participants and the initial parsimonious model are provided in the supplementary materials (Supplementary Table S1 and Table S2), the extended multivariable model (*N* = 350) offered the highest explanatory power (Nagelkerke *R*^*2*^ = 0.199) and excellent discriminative ability (AUC = 0.900). This comprehensive model demonstrated a significant fit over the intercept-only specification (LR χ^2^(22) = 44.687, *p* = 0.003) and showed no evidence of miscalibration (Hosmer–Lemeshow *p* = 0.858). The detailed multivariable analysis results presented in Table 4 revealed several key predictors of maternal concern: vaccine concerns emerged as the strongest predictor, with mothers reporting such concerns being 8.98 times more likely to experience concern (aOR = 8.98, 95% CI: 4.56–18.69, *p* < 0.001). Believing that the heel-prick procedure is painful also significantly increased the likelihood of maternal concern (aOR = 4.70; 95% CI: 2.18–10.94; *p* < 0.001), whereas those who were undecided about the procedure’s painfulness showed a similar but nonsignificant trend (aOR = 2.85; *p* = 0.059). Furthermore, compared with living in the city center, residing in a village was associated with a 3.18-fold increase in the odds of concern (aOR = 3.18; 95% CI: 1.10–9.32; *p* = 0.032), and knowing someone who refused the screening was marginally associated with increased concern (aOR = 3.20; *p* = 0.080). Other variables, including maternal age, education level, economic status, and religious or cultural influences, did not emerge as significant independent predictors in the final model (*p* > 0.05) (Table 4).

**Table 4:** Multivariable Firth-penalized logistic regression model for predictors of maternal concern toward neonatal heel-prick screening (*N* = 350)

Predictor	aOR (95% CI)	*p*
Age (years)	1.00 (0.94–1.07)	0.953
Education: medium (ref = low)	1.39 (0.68–2.88)	0.372
Economic Medium (ref = good)	1.81 (0.88–3.88)	0.110
Poor (ref = good)	1.64 (0.14–10.92)	0.647
Residence District (ref = city center)	1.64 (0.72–3.63)	0.233
Village (ref = city center)	3.18 (1.10–9.32)	0.032
Vaccine concern: Yes (ref = no)	8.98 (4.56–18.69)	< 0.001
I think heel-prick sampling is a painful procedure: yes (ref = no)	4.70 (2.18–10.94)	< 0.001
I think heel-prick sampling is a painful procedure: undecided (ref = no)	2.85 (0.96–8.46)	0.059
Knowing someone who refused heel-prick: yes (ref = no)	3.20 (0.86–10.86)	0.080
Religious/cultural influence: yes (ref = no)	1.69 (0.44–6.67)	0.439

The outcome was maternal concern toward neonatal heel-prick screening (binary). Estimates are aORs (95% CIs) from the Firth penalized logistic regression (logistf); *p* values are two-sided, and *p* < 0.001 is displayed as < 0.001. Reference categories are provided within predictor labels. Sample size: *N* = 350, 62 events, complete-case *n* = 350. The parsimonious model retained key covariates with improved stability relative to the extended specification (k_params = 13; EPV = 5.17). The overall fit was better than that of the null model (logLik_full =  − 147.780 vs null − 161.495; LR *χ*^2^(12) = 27.430; *p* = 0.007). The information criterion was AIC = 321.6 and BIC = 371.7; the explained variation was Nagelkerke *R*^2^ = 0.125. Discrimination remained good (AUC = 0.872), with Brier = 0.100. The calibration results were close to ideal (intercept =  − 0.064; slope = 1.065), and auxiliary calibration testing revealed no evidence of lack of fit (HL *χ*^2^(8) = 4.489; *p* = 0.811; 10 groups)

## Discussion

### Concern status and attitudes toward heel-prick screening

In this study, the relationships among postpartum mothers’ concerns, attitudes, and knowledge levels regarding newborn heel-prick screening were examined. Overall, the majority of mothers had a positive attitude toward the screening process. However, 17.7% of the participants reported experiencing concern related to the procedure, whereas 9.7% reported that they refused heel-prick blood sampling for their newborns.

In a study conducted in the Netherlands, the percentage of parents who refused heel-prick blood collection was 3% (16/515) in 2019 and 5% (32/647) in 2020. Furthermore, parents who refused were found to be more indecisive than those who participated [16]. Similarly, Botkin et al. reported that approximately 1.4% of mothers reported that they had declined newborn screening in a study conducted across three states in the USA; however, the authors noted possible confusion regarding the nature of the screening [9]. Newcomb et al. emphasized that mothers’ lack of knowledge about newborn screening during the postpartum period is common, and this situation is associated with concern and indecision, especially in groups with low socioeconomic status [17]. In a systematic review conducted by Carlton et al., the level of concern was evaluated as an indicator of the acceptability of screening tests [14]. In this study, 73.5% of the mothers who refused heel-prick blood collection reported experiencing concern (*p* < 0.001), indicating that concern plays a key role in indecision and refusal behavior. The present findings also suggest that the increasing refusal rates for heel-prick screening tests reported in the international literature are reflected in the Turkish population as well.

### Effects of rural life and sociodemographic factors

Our study clearly demonstrated the impact of rural residence on maternal concern. Mothers living in villages had a 3.18-fold greater likelihood of reporting concern. In addition, peer influence and social media emerged as significant external determinants. These findings suggest that in rural or socially closed communities, misinformation may spread more rapidly, thereby amplifying maternal concern. These results are consistent with the literature. A study conducted in India in 2025 reported that the majority of mothers with negative attitudes toward heel-prick blood sampling resided in rural areas, and this association was statistically significant [3]. Rural living conditions—characterized by low health literacy, limited access to healthcare professionals, and the influence of traditional or religious beliefs—may contribute to a deepening lack of accurate information. Collectively, these findings underscore the need to develop targeted educational and awareness-raising interventions, particularly in rural and socioeconomically disadvantaged regions.

### Role of religious and cultural factors

Mothers’ concerns related to the screening process significantly increased if they knew someone in their environment who had refused heel-prick blood collection for any reason or for religious/cultural reasons (43.2% and 50%, respectively). Among mothers who reported that heel-prick blood collection was contrary to their religious beliefs, 66.7% reported concern, and this association was statistically significant.

These findings indicate that mothers who refuse to have their babies’ heel-prick may be more prone to concern and rejection in an environment where awareness levels are low or where cultural/religious beliefs are influential. Our study revealed that parents’ attitudes toward newborn screening are shaped by their social environment, religious and cultural beliefs and level of knowledge. A study by van der Pal et al. revealed that parents who refused screening have an active religious belief (57% vs. 23%) and adopt an alternative/natural lifestyle compared with those who participated in screening [16].

### Vaccine concern and screening-related concerns

In our study, 42.5% of mothers with vaccine concerns and 25.7% of mothers who had not been vaccinated during childhood reported concern about neonatal heel-prick screening. In support of these findings, our extended multivariable regression analysis revealed that vaccine concern was the strongest independent predictor of maternal concern. Mothers who expressed such concerns were 8.98 times more likely to report concern regarding the screening procedure. These results suggest that vaccine concern is not merely a coexisting attitude but may also represent one of the major determinants of concern related to the screening process. Furthermore, our findings indicate that concerns about vaccines may influence attitudes not only toward immunization but also toward other preventive health practices, such as newborn screening. Consistent with this interpretation, van der Pal et al. reported that more than half of parents who refused heel-prick screening (57%, *n* = 27) did not plan to have their children receive routine childhood vaccinations [16]. In the literature, concerns about vaccines have been associated with doubts about the efficacy and safety of vaccines, as well as with religious and cultural beliefs [18–20]. In this context, similar factors may also contribute to increased concern regarding heel-prick screening. Therefore, coordinated communication strategies addressing both newborn screening and immunization may improve parental acceptance of preventive health services.

### Relationships between knowledge levels and concern regarding heel-prick screening

A substantial proportion of participants reported that they had not received adequate information about the heel-prick test. This lack of information was found to be associated with maternal concern. Insufficient information may lead to increased concern, lower acceptance, and a greater tendency among mothers to refuse screening. In our study, the majority of mothers who believed that the heel-prick test should be mandatory and who knew that it is performed to detect genetic and congenital disorders reported that they did not experience any concern. These findings suggest that as parents’ knowledge increases, their concern decreases. It is widely acknowledged in the literature that parents have insufficient knowledge about newborn screening. Studies have shown that very few parents remember that newborn screening is performed on their child and that even fewer parents adequately understand the purpose of the test [5, 13, 21]. When parents have adequate information about the screening process, their concern decreases, acceptance rates increase, and they exhibit more positive attitudes toward screening when appropriate counselling is provided [4, 12].

### Perception and concern related to heel-prick screening

In our study, a strong correlation was identified between mothers’ negative perceptions of neonatal heel-prick screening and their presence of concern. While subjective concerns regarding pain are common, our multivariable analysis revealed that the perception of the heel-prick procedure as “painful” independently increases the risk of high concern by nearly five times. van der Pal et al. reported that the most common sources of parental concern and reasons for refusing newborn screening programs in the Netherlands were “the procedure being painful,” “doubts about the reliability of the results,” and “uncertainties regarding how the data obtained from the samples are processed” [16, 23]. Another study indicated that negative experiences related to pain play a key role in parents’ decision to refuse the procedure [22]. In the systematic review conducted by Tluczek et al., the invasive nature of the procedure was emphasized as the main source of stress for parents [2]. Our study supports the idea that providing parents with accurate, clear, and sufficient information plays a critical role in reducing concern and refusal of the procedure.

The lack of information about test results and uncertainty regarding when they will be communicated increases parental concern. Previous studies have shown that parental concerns are most commonly associated with insufficient information, unclear test results and false-positive outcomes [2, 5, 6, 9, 21, 24]. Similarly, it has been reported that parents who are well informed experience less stress when confronted with false-positive results [8]. This suggests that parental concern is related not only to the procedure itself but also to the clarity and transparency of communication and information sharing.

### Clinical and public health implications

The present findings may have several clinical and public health implications. Healthcare professionals should consider assessing mothers’ general attitudes toward vaccination before or during counselling for neonatal heel-prick screening, as concerns about vaccines may indicate broader hesitation toward preventive health practices. Mothers expressing vaccine-related concerns may benefit from more individualized counselling rather than standard procedural information alone. Such counselling should focus on addressing specific concerns, explaining the purpose and safety of heel-prick screening, and improving parental understanding of the potential benefits of early diagnosis.

From a public health perspective, targeted educational strategies may be particularly important in rural areas, where concerns and misinformation may be more common. Family physicians, midwives, and other primary healthcare professionals can play key roles in explaining the purpose, safety, and importance of newborn screening. Educational materials, including brochures, visual materials, and social media-based content, may also support health literacy. In addition, clear and timely communication of screening results to families may help strengthen their trust in newborn screening programs and improve their participation.

### Strengths and limitations

This study has several limitations. First, its cross-sectional design precludes causal inference and does not allow for the determination of temporal directionality between maternal perceptions and concern. Second, the sample was drawn from a single hospital and represents a specific regional population, which may limit the generalizability of the findings to the national level or to different sociocultural contexts. Third, because the data were collected through face-to-face interviews, social desirability bias cannot be excluded. Fourth, maternal concern was assessed using a researcher-developed questionnaire rather than a validated psychometric instrument; therefore, the findings reflect screening-specific self-reported concern, and measurement limitations should be considered when the results are interpreted. Fifth, although participants were recruited from the obstetric ward, neonatal intensive care unit, and neonatal outpatient clinic, the specific clinical setting in which each questionnaire was administered was not recorded as a separate analytical variable. Therefore, subgroup analysis according to clinical setting could not be performed. Since maternal emotional responses may differ according to the newborn’s clinical condition, particularly among mothers of infants followed in the neonatal intensive care unit, this should be considered when the findings are interpreted.

Despite these limitations, the study has several strengths. To our knowledge, this is among the few studies in Türkiye to comprehensively and simultaneously assess mothers’ knowledge, attitudes, and concerns regarding newborn heel-prick screening within an integrated analytical framework. The methodological strength of the study is supported by a relatively large final sample size (*N* = 350), standardized data collection under controlled single-center conditions, and rigorous multivariable analyses, including Firth penalized logistic regression. Importantly, by incorporating the emotional dimension of parental experiences, the study extends beyond descriptive assessments and provides a nuanced perspective on the acceptability and psychosocial dimensions of newborn screening programs.

## Conclusion

This study revealed that maternal concern about neonatal heel-prick screening was associated with concerns about the use of vaccines, rural residence, perceived pain, insufficient knowledge, and negative perceptions of the procedure. These findings suggest that clear communication, culturally sensitive counselling, and targeted education for mothers with broader concerns about preventive health practices may help reduce hesitation and improve participation in newborn screening programs. However, given the cross-sectional design and the use of a researcher-developed questionnaire, these findings should be interpreted as associations rather than causal relationships.

## Supplementary information

Below is the link to the electronic supplementary material.

ESM 1 (DOCX 20.7 KB)

## Data Availability

The datasets generated and/or analyzed during the current study are not publicly available due to privacy and ethical restrictions but are available from the corresponding author on reasonable request.
