# Supplementary material for: Maternal concern and refusal toward neonatal heel-prick screening: a cross-sectional survey from Türkiye
Source: Eur J Pediatr. 2026 May 7;185(6):355. doi: 10.1007/s00431-026-07025-y (PMC13149568; doi:10.1007/s00431-026-07025-y)
Supplement: Supplementary file 1 — (DOCX 20.7 KB) [file 431_2026_7025_MOESM1_ESM.docx]

# Supplementary Tables

# Table S1 Primary Multivariable Firth Penalized Logistic Regression Model For Maternal Concern Toward Neonatal Heel-Prick Screening (N = 350)

| **Predictor** | **aOR (95% CI)** | **p** |
| --- | --- | --- |
| **Age** (years) | 1.00 (0.94-1.07) | 0.967 |
| **Education**: Medium (ref=Low) | 1.40 (0.68-2.90) | 0.361 |
| **Economic** |  |  |
| Medium (ref=Good) | 1.78 (0.87-3.79) | 0.118 |
| Poor (ref=Good) | 1.72 (0.15-11.37) | 0.615 |
| **Residence**  District (ref=City center) | 1.66 (0.73-3.69) | 0.224 |
| Village (ref=City center) | 3.14 (1.09-9.22) | 0.035 |
| **Vaccine concern:** Yes (ref=No) | 9.98 (5.12-20.57) | <0.001 |
| **Knowing someone who refused heel-prick:** Yes (ref=No) | 4.50 (1.87-10.95) | <0.001 |
| **I think heel-prick sampling is a painful procedure.** Yes (ref=No) | 4.55 (2.13-10.47) | <0.001 |
| **I think heel-prick sampling is a painful procedure.** Undecided (ref=No) | 2.67 (0.91-7.78) | 0.074 |

**Note.** The outcome was Maternal Anxiety Toward Neonatal Heel-Prick Screening (binary). Estimates are adjusted odds ratios (aORs) from Firth penalized logistic regression (logistf) with 95% confidence intervals and two-sided p-values; p < 0.001 is reported as <0.001. Reference categories are shown in the predictor labels. Sample size was N = 350 with 62 events (complete-case n = 350). Model support and global fit were: EPV = 6.20, logLik_full = −149.392 (null = −161.495), and the model improved fit over the intercept-only model (LR χ²(10) = 24.205, p = 0.007). Comparative information criteria were AIC = 320.8 and BIC = 363.2; explained variation was modest (Nagelkerke R² = 0.111). Discrimination was good (AUC = 0.871) with overall probability accuracy Brier = 0.101. Calibration was close to ideal (intercept = −0.056; slope = 1.056). The Hosmer–Lemeshow statistic (10 groups) did not indicate lack of fit (HL χ²(8) = 4.607; p = 0.799), interpreted as an auxiliary calibration check.

**Table S2 Extended Multivariable Firth Penalized Logistic Regression Model For Maternal Concern Toward Neonatal Heel-Prick Screening (N = 350)**

| **Predictor** | **aOR (95% CI)** | **p** |
| --- | --- | --- |
| **Age** (years) | 1.02 (0.95-1.10) | 0.523 |
| **Education:** Medium (ref=Low) | 1.18 (0.53-2.65) | 0.678 |
| **Economic**  Medium (ref=Good) | 1.67 (0.77-3.81) | 0.198 |
| Poor (ref=Good) | 2.17 (0.15-15.44) | 0.511 |
| **Residence:**  District (ref=City center) | 1.74 (0.72-4.09) | 0.212 |
| Village (ref=City center) | 4.47 (1.42-14.49) | 0.011 |
| **Chronic disease:** Yes (ref=No) | 1.02 (0.38-2.55) | 0.973 |
| **Received information:** Yes (ref=No) | 1.09 (0.53-2.30) | 0.813 |
| **Timing of information:** Late (ref=Early) | 0.81 (0.36-1.87) | 0.621 |
| **Influence of illness history:** Yes (ref=No) | 0.86 (0.20-3.22) | 0.823 |
| **Knowing someone who refused heel-prick:** Yes (ref=No) | 3.48 (0.88-12.40) | 0.074 |
| **Religious/cultural influence:** Yes (ref=No) | 1.52 (0.39-6.35) | 0.553 |
| **Info source:**  Healthcare staff (ref=No) | 0.67 (0.30-1.52) | 0.333 |
| Social media (ref=No) | 2.66 (1.16-6.15) | 0.021 |
| TV (ref=No) | 0.50 (0.08-2.39) | 0.401 |
| Peers (ref=No) | 4.61 (1.89-11.63) | <0.001 |
| Scientific sources (ref=No) | 1.28 (0.42-3.77) | 0.655 |
| **Vaccine concern:** Yes (ref=No) | 8.78 (4.28-19.33) | <0.001 |
| **I think heel-prick sampling is a painful procedure.** Yes (ref=No) | 4.84 (2.10-12.20) | <0.001 |
| **I think heel-prick sampling is a painful procedure.** Undecided (ref=No) | 2.04 (0.62-6.57) | 0.233 |

**Note.** The outcome was Maternal Anxiety Toward Neonatal Heel-Prick Screening (binary). Results are aORs (95% CIs) from Firth penalized logistic regression (logistf) with two-sided p-values; p < 0.001 is shown as <0.001. Reference categories are indicated in the predictor labels. Analytic sample: N = 350, 62 events, complete-case n = 350. Data support was limited (EPV = 2.82), so coefficients should be interpreted with attention to stability despite penalization. Model fit relative to the intercept-only model was significant (logLik_full = −139.151 vs null −161.495; LR χ²(22) = 44.687, p = 0.003). Information criteria were AIC = 324.3 and BIC = 413.0; explained variation increased (Nagelkerke R² = 0.199). Discrimination was excellent (AUC = 0.900) with improved probabilistic accuracy (Brier = 0.088). Calibration metrics suggested reasonable agreement (intercept = −0.113; slope = 1.129). The Hosmer–Lemeshow test (10 groups) did not suggest miscalibration (HL χ²(8) = 3.995; p = 0.858), used here as a supportive diagnostic.
